# Resilience of young adults living with mental disorders in the City of Tshwane, South Africa

**DOI:** 10.4102/hsag.v30i0.2857

**Published:** 2025-03-13

**Authors:** Nok’khanya F. Hadebe, Richard M. Rasesemola

**Affiliations:** 1 Department of Emergency Medical Care, Faculty of Health Sciences, University of Johannesburg, Johannesburg, South Africa; 2 Department of Nursing, Faculty of Health Sciences, University of Johannesburg, Johannesburg, South Africa

**Keywords:** young adults, resilience, purposefulness, confidence, self-esteem

## Abstract

**Background:**

The challenges that young adults encounter today present greater risks to their mental wellbeing compared to those experienced by previous generations. For those young adults living with mental health disorders, they suffer even more when having to deal with its burdens. Many of these young adults face floods of negative and powerful emotions, discrimination and isolation; however, they are likely to cope well with these difficult situations if they are resilient.

**Aim:**

This study purported to report on resilience among young adults living with mental disorders in the City of Tshwane.

**Setting:**

The study was conducted in Atteridgeville in the City of Tshwane, Gauteng province in South Africa.

**Methods:**

A qualitative exploratory and descriptive research was conducted among 10 purposefully sampled young adults aged between 18 years and 34 years old. Data were collected using individual, semi-structured interviews in primary healthcare clinics and analysed using Tesch’s method.

**Results:**

Two themes, namely, *harnessing purpose in building resilience* and *confidence enhances positive interactions* along with four related sub-theme emerged from the results. The themes indicated how a sense of purposefulness and confidence help young adults living with mental health disorders build resilience.

**Conclusion:**

For young adults to be resilient, approaches such as psychotherapy for treating mental health issues need to incorporate strategies to develop a sense of purpose and confidence.

**Contribution:**

This study elucidated the role of purposefulness and confidence in building resilience, and further provided some suggestions on strategies that could be used to build resilience in conjunction with medical management of mental health disorders among young adults.

## Introduction

Mental health disorders are generally characterised by clinically significant disturbances in an individual’s cognition, emotion regulation or behaviour (World Health Organization 2022). These clinical disturbances reflect dysfunction in the psychological, biological or developmental processes underlying mental functioning and are often accompanied by discrimination (World Health Organization 2022). Young adults often experience prejudice and discrimination, and they suffer even more when living with mental health disorders and having to deal with their burdens which limit their capacity to acquire the skills to build resilience (Hadebe & Ramukumba 2020; Kirkbride et al. 2024).

There is no specific cause of mental health disorders; however, the development and susceptibility to mental health disorders are linked to risk factors such as genetics, biological and environmental factors (World Health Organization 2022). Craig et al. (2022) reported on some factors related to the development of mental health disorders. Factors such as socioeconomic challenges as a result of the history of colonialism and apartheid in South Africa led to high levels of income inequality and unequal access to healthcare (including mental healthcare services), education and economic opportunities; the lack of employment opportunities which leads to stress, anxiety and depression; adverse childhood experiences which have strong links with depression and anxiety, as well as traditional gender roles that often lead men to refrain from expressing their emotions and neglect their own feelings and for others. Moreover, immense pressure to succeed academically and professionally may exacerbate feelings of inadequacy and stress.

Other factors related to the development and susceptibility to mental health disorders include coronavirus disease 2019 (COVID-19) pandemic, which significantly increased the number of people living with mental health disorders from 2020 (World Health Organization 2022). The advent of the COVID-19 pandemic affected many young adults’ mental health. Isolation, loneliness, loss of a sense of purpose and a lack of confidence to deal with challenges of not being able to participate in economic activities are some of the factors that led to increased number of young adults to develop mental health disorders (Khan et al. 2022). Bansal et al. (2024) also reported on the income inequality, political instability and factors related to the internet such as cyberbullying as having strong links to the rapid shift in susceptibility to mental health disorders in young adults.

Globally, up to 25% of young adults suffer from some form of mental health disorder (McGrath et al. 2023). In South Africa, up to 44% of young adults are reportedly living with some form of mental health disorder (Bantjes et al. 2019) with mood disorders and depression, anxiety and substance abuse disorders being some of the most common ones (Sorsdahl et al. 2023). These disorders tend to develop from as early as 14 years of age, both in South Africa and around the world (Bantjes et al. 2019). Despite the high figures of mental health disorders in South Africa, almost 75% of people with these disorders do not have access to any form of mental healthcare (Sorsdahl et al. 2023; South African College of Applied Psychology 2019). According to Sorsdahl et al. (2023), because of poor prioritisation of mental health services and a lack of resources such as qualified personnel, special health programmes and infrastructure for mental health services in South Africa, young adults living with mental health disorders may find themselves lacking resilience.

Resilience refers to the process of adapting well in the face of adversity, trauma, tragedy, threats or even significant sources of stress (American Psychological Association 2024) which includes a combination of personal characteristics and some learned skills. Others refer to resilience as the capacity to adapt, cope, rebound, withstand, grow, survive and define a new sense of self through situations of adversity, including mental health disorders (Shrivastava & Desousa 2016). No matter how resilience is defined, it has been shown to have a strong connection with mental wellbeing, overall wellbeing and greater life satisfaction (Konaszewski, Niesiobędzka & Surzykiewicz 2021).

The American Psychological Association (2024), among others, suggests that everyone has the ability to be resilient if they can integrate the tenets of resilience in mental health such as: (1) *adaptability,* where one is flexible and develops coping strategies when dealing with situations beyond their control, (2) *social resources,* where one can access quality social support by building good relationships with others, seeking support when needed and having resources that allow them to overcome difficult situations rather than try to cope on their own, (3) *personal skills* including self-esteem and communication skills which relates to having feelings of having control over challenging situations as well as competence and effectiveness in coping with stressful situations, (4) *purposefulness*, which helps one to put things in perspective when facing stresses through challenging times.

For young adults, who are often faced with floods of negative and powerful emotions, they are likely to cope well when confronted with challenges and difficult situations if they are resilient (Li et al. 2023). Currently, young adults must deal with challenges, demands and expectations that their peers, some generations ago never had to deal with (Li et al. 2023). Hence, they are vulnerable to the development of mental health disorders because of these challenges, experiences and circumstances (World Health Organization 2024). This study intended to look at how young adults living with mental health disorders in the City of Tshwane can build resilience despite the challenges associated with living with these disorders. Therefore, the aim of this research study was to explore and describe the resilience of young adults living with mental health disorders in the City of Tshwane.

## Research methods and design

A qualitative, exploratory and descriptive design was adopted for this study to explore and gain an insight and understanding of how the young adults living with mental health disorders in the City of Tshwane manage to develop and sustain resilience. Data collection was conducted through semi-structured interviews.

### Study setting

The study was conducted at two primary healthcare clinics offering mental healthcare service in Atteridgeville, a township in the West of Pretoria Central Business District in the City of Tshwane, in Gauteng province of South Africa. Atteridgeville township, like many other townships in South Africa, is characterised by increasing prevalence of substance use disorders (Kroukamp 2018), high levels of crime and increasing youth unemployment rates (Department of Statistics 2023). The mental health care services in the City of Tshwane are provided through regional hospitals and district hospitals, community health centres, primary healthcare clinics, satellite service units and public rehabilitation centres. The private psychiatric hospitals and private rehabilitation centres are also accessible for those who have medical aid cover or can afford the services.

### Population and sampling

The population for this study included young adults, aged between 18 years and 34 years, living with mental health disorders residing in Atteridgeville in the City of Tshwane. The criteria for inclusion in the study were those young adults living with mood disorders, anxiety disorders and schizophrenia who were stable and able to provide consent. Those with substance abuse and those who were not able to provide consent were not included in the study. Purposive sampling, in accordance with the inclusion and exclusion criteria of the study, was used in this study. Primary healthcare clinics, where mental healthcare services are offered, were visited by the researcher who is a registered Psychiatric Nurse, to identify those individuals who fit the inclusion criteria and were willing to participate.

### Data collection

Primary healthcare clinics offering mental healthcare services in Atteridgeville were approached, and permissions were granted by the clinics’ operational managers. Potential participants who were identified from the primary healthcare clinics were approached for participation. Appointments for interviews, including the dates and times convenient for the participants were set, and interviews took place at the clinics in a separate room. Semi-structured interviews were conducted, and the researcher began each interview by asking: *Could you share with me how you cultivate resilience when facing challenges?* The question was followed by more probing questions. Each interview lasted between 30 min and 45 min and was recorded on an audio recording device and later transcribed verbatim. A total of 10 interviews were conducted, and the sample size was determined by data saturation where adding more participants or new data to the study yielded redundant information. To ensure that saturation is reached, which occurred at the seventh interview, the researcher went beyond the point of saturation by conducting three more interviews to make sure no new major concepts emerged.

### Data analysis

Data analysis was done after each interview. Data were analysed using Tesch’s method which involves categorising verbatim data into themes (Tesch 1990). Interview transcripts were read, and initial notes of ideas that emerged while trying to get the meaning in the information were taken. Similar topics identified from the notes made on the transcripts were arranged in groups, and preliminary themes were developed from the notes. Data were reassessed by the researcher and study supervisor to check if there were new themes or codes emerging. Data material belonging to each theme were put together in one place to come up with the final themes.

### Measures of trustworthiness

Trustworthiness in this study was ensured by adhering to the criteria by Lincoln and Guba (1985) including credibility, transferability, confirmability and dependability. Credibility was ensured by the use of prolonged engagement, reflexibility and triangulation (Lincoln & Guba 1985). The researcher spent time in the field building rapport with the participants. An independent coder was used to ensure that researcher’s personal biases and preconceptions do not influence the outcome of the study. Triangulation was ensured by using multiple data sources including interviews and observation notes to cross-verify findings.

To ensure transferability, the researcher provided a full and in-depth description of the study’s context, participants and research design to enable readers to judge the applicability and relevance of findings to their own context (Stahl & King 2020). Methodological documentation and audit trails, where the researcher thoroughly documented each step of the research process, were also conducted to ensure dependability. The researcher further used member checking and reflexive journaling to ensure confirmability. Participants were involved in the verification process to ensure that their viewpoints and experiences were accurately represented and to provide an opportunity for participants to validate or offer corrections to the interpretations. A reflexive journal that was written consistently after each interview and through each step of the study included researcher’s personal thoughts, biases and reflections.

### Ethical considerations

The study was approved by the Faculty Research Ethics Committee of the Tshwane University of Technology (FRCE 2017/10/08 [SCI] [2]) and Gauteng Department of Health (GP_201711_015). Informed consent, which requires participants to voluntarily confirm their willingness to participate in a study after being informed of all aspects of the research relevant to their participation, was obtained from each participant before they participated in the study (Varkey 2021). Autonomy, which requires participants to have a full understanding of what is being asked of them to make a reasoned judgement to participate, without influence or coercion (Varkey 2021), was ensured when participants were informed about all aspects of the study through an information leaflet. To ensure beneficence and minimise harm, the researcher continuously assessed and ensured the participants’ wellbeing, including physical, psychological and emotional wellbeing. Confidentiality was upheld by ensuring that the interviews occurred in a private space, while anonymity was ensured by protecting participants’ identities, securing all data related to the study in a password-protected folder in the researcher’s personal computer and using pseudonyms, thus, ensuring that no link between the individual identities of the participants to the research can be made during reporting.

## Results

Table 1 reflects participants’ demographic information. There were 10 participants with ages ranging between 19 years and 34 years; 7 of the 10 participants were males. All participants were African, and their educational background ranged from Grade 11 to a diploma. Two participants were scholars, three were employed and five were unemployed.

**TABLE 1:** Demographic information (*N* = 10).

Gender	*n*	Age (years)	*n*
Male	7	19–24	3
		25–29	3
Female	3	30+	4

As reflected in Table 2, two themes, namely, *harnessing purpose in building resilience* and *confidence enhances positive interactions* were generated from the participants’ experiences of how they managed to cultivate resilience when facing challenges. Participants’ descriptions in these themes expressed how embracing optimism and a desire for a normal life, and fostering determination and hope for positive prospects in life helped them gain a sense of purposefulness. The participants further expressed and described how self-doubt and paranoia affected their interactions with others and how self-esteem eased positive engagement with others.

**TABLE 2:** Themes: Harnessing purpose in building resilience, and confidence enhances positive interactions.

Themes	Sub-themes
1. Harnessing purpose in building resilience.	1.1 Embracing optimism and desire for a normal life. 1.2 Fostering determination and hope for positive life.
2. Confidence enhances positive interactions.	2.1 Self-doubt and paranoia often affected interaction with others. 2.2 Self-esteem eased positive engagement with others.

### Theme 1: Harnessing purpose in building resilience

Some participants expressed a clear sense of purpose, indicated by their drive to persist, and achieve their goals in the face of setbacks. According to the participants, having a desire for a normal life, being optimistic, having determination and hope or prospect for a positive life made them resilient.

#### Sub-theme 1.1: Embracing optimism and desire for a normal life

According to the participants, a sense of optimism and a desire to live ‘a normal life’ despite the challenges of living with a mental health disorder made them develop resilience. One participant who was diagnosed with depression expressed that their life will be normal because of their belief in God:

‘You see, if I get mercy of God, I want to have … I like to hear people say everything is possible you know, but everything is not always possible but it’s possible. So, I just want to achieve good things you see, like having a good family, wife, kids, my own house, and a job so that I can be okay and live a normal life. That’s all I want.’ (Male, 31 years old)

Another participant who was diagnosed with mood disorders expressed feeling tired of being on medical treatment for her disorder and the stigma associated with it. The participant expressed a desire to lead a normal life where there would not be a need for them to disclose their own disorder and having people feeling sorry for them:

‘No one wants to live the kind of life I live, I also want to be normal, like any other 28-year-old lady, not having to take medication to control how I feel, not having to disclose anything to anyone because of fear of judgement [*long pause*], and not having anyone feeling sorry for me.’ (Female, 28 years old)

#### Sub-theme 1.2: Fostering determination and hope for positive prospects in life

This sub-theme is about people finding meaning and purpose, encouragement, aspirations and belief in their own ability to overcome challenges despite living with mental health disorders. Participants indicated a sense of determination, recognised their own potential and expressed the ability to cultivate a positive mindset by setting goals and working towards them:

‘I just have to make sure I work hard and concentrate at school so that after my diploma I can get internship and start saving towards my goals.’ (Male, 19 years old)

‘I want to see myself having a big shop and I want to see myself having a family [*brief pause*] I try to save a little from the commission I make from selling.’ (Female, 34 years old)

Despite a feeling of not having achieved what their peers have achieved, another participant indicated a positive mindset by setting goals, working towards them and having hope to achieve them:

‘My goal is to own an IT company, I want to have my own house, cars, and I want to start a family you see? My friends have made it in life, and I am still trying to get a permanent job, so I still have to catch up but I will get there.’ (Female, 34 years old)

### Theme 2: Confidence enhances positive interactions

This theme is about how confidence plays a crucial role in enhancing positive interactions among people living with mental health disorders. Participants reflected on how they navigate interactions with other people. Confidence empowered them to navigate social situations with greater ease, promoted self-worth and belonging, while the lack of it led to self-doubt and paranoia.

#### Sub-theme 2.1: Self-doubt and paranoia often affected interaction with others

Self-doubt and paranoia had significant effects on the ability of people living with mental health disorders to interact with others. The participants constantly questioned their own self-worth, and displayed difficulty expressing themselves to others. Paranoia created barriers in interactions in one participant who expressed hesitancy in social situation, mistrust and suspicions towards other people:

‘Sometimes I can see the way people look at me. It’s like I’m different … I think they do not like me much I don’t know how to tell you … but I can’t do anything about that. It’s okay I guess [*looking down, not maintaining eye contact*].’ (Male, 23 years old).

One participant displayed self-doubt and a lack of confidence when they indicated an inability to express themselves and having communication barriers, and feelings of isolation, which contribute to the feelings of isolation and a lack of self-worth:

‘[*Sigh and long pause*] I just feel … I just feel awkward. I just feel like the world is against me, so I just feel … you see, some of the things I cannot tell you. It just makes me feel useless actually.’ (Male, 32 years old)

#### Sub-theme 2.2: Self-esteem eased positive engagement with others

This sub-theme indicates how having a sense of self-esteem enhances a positive interaction with others among people living with mental health disorders. Those individuals who expressed being confident reported eased social engagements, expressed themselves openly and expressed the ability to make meaningful connections.

One participant indicated positive attributes and recognised their own strengths, with behaviours such as good interactions with other people, recognised their self-worth and belonging and reflected on the communication abilities:

‘I’m a confident person, I’m not shy. I developed a lot relationships with many people, I am good in communicating with people, and I am good with working with them and yeah, stuff like that.’ (Male, 31 years old)

Another participant indicated how having confidence enhances feelings of self-worth and fosters a sense of equality with a greater ease of acceptance and understanding from others:

‘No one makes me feel less about myself. We have this understanding, it’s a give and take and we are equal at the table, then we live well with each other [*Long pause*], and yeah, I can say my confidence is fine because if I say I want to do something I do it.’ (Male, 29 years old)

## Discussion

The findings in this study indicated that participants were resilient because they had a sense of purpose, a drive to achieve their goals and a sense of optimism in life despite the challenges of living with mental disorders. Resilience is having a clear sense of purpose, clear values, drive and direction that help individuals to persist and achieve in the face of challenges (American Psychological Association 2024). Generally, purposefulness has a positive effect in building greater resilience and one’s ability to adapt positively after exposure to negative events (South African College of Applied Psychology 2019). Purposefulness involves identification and commitment to one’s life ambitions and ability to organise one’s goals (Hill, Edmonds & Hampson 2019). These attributes have been known to help guide in selecting one’s daily behaviours and activities that match one’s long-term aims. The obvious advantages of engagement in activities for overall wellbeing and health appear to be the likely explanation for why individuals with greater purposefulness experience greater health benefits (Hill et al. 2019).

In mental healthcare, purposefulness strengthens resilience and prevents relapse in people living with mental health disorders (Yamashita, Yoshioka & Yajima 2021). The significance of purposefulness lies in its strong ability for behavioural activation and subsequent positive changes, and reduced symptoms among some people living with mental health disorders (Boreham & Schutte 2023). There are reports of purposefulness as a reliable and significant predictor of future occurrence of mental health disorders, where low levels of purposefulness significantly predicted the likelihood of developing some mental health disorders in future (Öcal et al. 2022). Although improvements for a sense of purposefulness among people living with mental health disorders came after medical treatment, there is sufficient evidence to support a relationship between lesser purposefulness and more symptoms of mental health disorders. This study supports the notion that having a sense of purposefulness, and engaging in daily activities that indicate a drive to set and achieve goals allow one to develop resilience despite the challenges of living with mental health disorders. Therefore, assisting people living with mental health disorders to develop a sense of purpose is crucial in mental healthcare because of its significant association with reduced levels of symptoms in mental health disorders.

The findings further indicated that participants had a sense of optimism. Optimism is a way of thinking and involves expecting positive results from life, an important attribute of resilience (Cheng & Chen 2024). Optimism influences mental and physical wellbeing by the promotion of a healthy lifestyle and behaviours, problem-solving capacity, and flexibility (Conversano et al. 2010). In his 1710 work, *Théodicée*, the German philosopher and mathematician, Gottfried Wilhelm Leibniz raised arguments for the doctrine that led to optimism to be commonly referred as the best of all possible worlds (Look & Belaval 2024). Thus, optimism reflects one’s favourable view about their future and leads to a general positive expectation – a thinking ability crucial to construct purposeful goals. According to Lee et al. (2022), optimism is a crucial element for purposefulness; and the link between purposefulness and mental wellbeing has already been established in this article. Like purposefulness, optimism is related to fewer symptoms of mental health disorders and higher levels of mental wellbeing (Carver & Scheier 2019).

The role of optimism in mental wellbeing is further expressed by Boreham and Schutte (2023), whereby, they indicate that optimism influences mental wellbeing by promoting adaptability and problem-solving. Other positive effects of optimism, according to Carver and Scheier (2019), may relate to the higher likelihood of adopting health promoting behaviours and coping strategies that enable better psychic adjustment for people living with mental disorders. Other researchers including Boreham and Schutte (2023), Conversano et al. (2010) and Schug et al. (2021) assert that optimism has an inversely proportional relationship with symptoms of some mental health disorders. This indicates that a higher level of optimism will lead to lower levels of symptoms in people living with mental health disorders, while increasing symptoms of mental health disorders may reduce the optimism. Thus, using psychotherapy and psychotropics as tools to manage mental health disorders should not only focus on alleviating symptoms but should also emphasise on fostering optimism. On the other hand, increased levels of optimism would not just lead to greater resilience but fewer symptoms of mental health disorders.

The findings in this study also indicated that participants’ belief in God gave them hope and ambitions to lead a productive life and helped them build resilience. Literature exists to highlight positive general health benefits of religious belief (Larson et al. 1992). In one study that explored interpretations of problems, coping strategies and help-seeking behaviour of black Christians attending Pentecostal churches in the United Kingdom, believing in God (faith in God) emerged as pivotal to mental health stability and wellbeing and was considered more effective and appropriate than therapy in some cases among people living with mental health disorders (Burrell 2019). Larson et al. (1992) also reported a positive correlation between various dimensions of religious commitment such as believing in God and mental wellbeing. Altruism, gratitude, positive cognitive appraisals, increased social support, healthy lifestyles and positive coping strategies, among others, in religious groups were reported as some of the possible mediating factors in the links between religion and mental wellbeing (Burrell 2019). However, Father Billy Swan, the Priest of Diocese of Ferns, who advocates for churches to be involved in the treatment of mental health disorders to allow holistic approach to mental healthcare, reports that faith in God is not a substitute for medical management of mental health disorders (Swan 2018). Therefore, the incorporation of religion, faith or spirituality in psychological interventions such as counselling and psychotherapy in mental healthcare should be considered to increase resilience among people living with mental health disorders.

Hope, which participants managed to harness because of their belief in God and is directed more towards the future is known as ‘a state of positive motivation based on three components: objectives (goals to be achieved), pathways (planning to achieve these goals), and agency (motivation directed toward these objectives)’ (Snyder 1991). Hope also has a strong association with purposefulness and resilience, and other several psychosocial processes and outcomes, including positive affect, emotional adjustment and illness-related coping, greater life satisfaction, enhanced perceptions that life is meaningful, a higher sense of purpose in life, quality of life and social support (Torales et al. 2024). During the COVID-19 pandemic, hope safeguarded people living with mental health disorders against symptoms of depression (Senger 2023).

The results further indicated the importance of confidence in resilience for people living with mental health disorders. Confidence, which is a belief in oneself and one’s abilities (Jekauc 2023) and self-esteem, which speaks about one’s opinion about oneself, one’s worth and one’s perception of one’s value as a person (Yanal 1987), made it easy for people living with mental health disorders to realise their own strengths and interact with others to establish social networks and build resilience. A lack of confidence, on the other hand, made participants have self-doubt, which made them paranoid and isolated. Confidence has a positive relationship with resilience, meaning the more confident people with mental health disorders are, the more positive they feel, leading to increased resilience and a reduced chance of succumbing to challenges associated with living with mental health disorders (Echezarraga et al. 2018). Low confidence and self-esteem might result in people living with mental health disorders feeling, among other things, ignored, excluded, unimportant and unloved (Yanal 1987), just as reflected in the findings. This means that those participants who lacked confidence also lacked resilience. Liu et al. (2021) also reflect that good self-esteem, which is also about viewing oneself from a positive perspective, manifested by more self-confidence, self-improvement and an ability to change the situation and better cope with various problems and stressors, is a protective factor for mental health and psychological functioning. Therefore, through cognitive behavioural therapy, people living with mental health disorders should be encouraged to focus on improving self-esteem and confidence to build resilience. By identifying negative thought patterns such as self-doubt and paranoia which often affect interaction with others, people living with mental health disorders can learn to challenge them, thus building a foundation for resilience and enabling them to navigate obstacles more effectively.

### Limitations

The study revealed an important role that purposefulness, optimism, determination, hope, religion and self-confidence and self-esteem play in building resilience among people living with mental health disorders. However, the study was qualitative and contextual, using purposive sampling of only 10 participants; therefore, the findings generated from this study cannot be generalised to other contexts.

### Recommendations

Several recommendations emanated from this study as follows:

- To build resilience among people living with mental health disorders, mental healthcare practitioners should focus on assisting people living with mental health disorders build a sense of purposefulness.
- The use of psychotherapy and psychotropics as tools to manage mental health disorders should incorporate activities that emphasise on fostering optimism instead of just focusing on alleviating symptoms of people living with mental health disorders.
- Incorporating religion, faith or spirituality in psychological interventions such as counselling and psychotherapy in mental healthcare should be considered to increase resilience among people living with mental health disorders.
- Cognitive behavioural therapies for people living with mental health disorders should also focus on improving self-esteem and confidence to build resilience and challenge negative thought patterns to enable them to navigate obstacles more effectively.

## Conclusion

Young adults face more challenges and demands that put strain on their mental health, which predisposes them to mental health disorders. For those already living with mental health disorders, discrimination, and loss of hope because of a lack of opportunities may further worsen their prospects of being a valuable member of the society, leading to low self-esteem, self-doubt, paranoia and ultimately loss of resilience. However, purposefulness and confidence play a role in promoting resilience among young adults living with mental health disorders.

## References

[CIT0001] American Psychological Association, 2024, *Resilience*, viewed 12 May 2024, from https://www.apa.org/topics/resilience.

[CIT0002] Bansal, S., Garg, N., Singh, J. & Van Der Walt, F., 2024, ‘Cyberbullying and mental health: Past, present and future’, *Frontiers in Psychology* 14, 1279234. 10.3389/fpsyg.2023.1279234 38288359 PMC10823540

[CIT0003] Bantjes, J., Lochner, C., Saal, W.D., Roos, J., Taljaard, L., Page, D. et al., 2019, ‘Prevalence and sociodemographic correlates of common mental illnesses among first-year university students in post-apartheid South Africa: Implications for a public mental health approach to student wellness’, *BMC Public Health* 19, 922. 10.1186/s12889-019-7218-y 31291925 PMC6617861

[CIT0004] Boreham, I.D. & Schutte, N.S., 2023, ‘The relationship between purpose in life and depression and anxiety: A meta-analysis’, *Journal of Clinical Psychology* 79(12), 2736–2767. 10.1002/jclp.23576 37572371

[CIT0005] Burrell, R., 2019, ‘The Black Majority Church: Exploring the impact of faith and a faith community on mental health and well-being’, Other thesis, Middlesex University/Metanoia Institute.

[CIT0006] Carver, C.S. & Scheier, M.F., 2019, ‘Optimism’, in M.W. Gallagher & S.J. Lopez (eds.), *Positive psychological assessment: A handbook of models and measures*, 2nd edn., pp. 61–76, American Psychological Association, Washington, DC.

[CIT0007] Cheng, C. & Chen, S., 2024, ‘Unmasking resilience in the “new normal”: Coping with unprecedented stressors amid Covid-19’, *Current Opinion in Behavioural Sciences* 55, 101346. 10.1016/j.cobeha.2023.101346

[CIT0008] Conversano, C., Rotondo, A., Lensi, E., Della Vista, O., Arpone, F. & Reda, M.A., 2010, ‘Optimism and its impact on mental and physical well-being’, *Clinical Practice and Epidemiology in Mental Health* 14(6), 25–29. 10.2174/1745017901006010025 PMC2894461 20592964

[CIT0009] Craig, A., Rochat, T., Naicker, S.N., Mapanga, W., Mtintsilana, A., Dlamini, S.N. et al., 2022, ‘The prevalence of probable depression and probable anxiety, and associations with adverse childhood experiences and socio-demographics: A national survey in South Africa’, *Frontiers in Public Health* 10, 986531. 10.3389/fpubh.2022.986531 36388391 PMC9650309

[CIT0010] Department of Statistics, 2023, *Beyond unemployment – Time-related underemployment in the SA labour market*, viewed 13 May 2024, from https://www.statssa.gov.za/?p=16312.

[CIT0011] Echezarraga, A., Calvete, E., González-Pinto, A.M. & Las Hayas, C., 2018, ‘Resilience dimensions and mental health outcomes in bipolar disorder in a follow-up study’, *Stress and Health* 34(1), 115–126. 10.1002/smi.2767 28639427

[CIT0012] Hadebe, N.F. & Ramukumba, T.S., 2020, ‘Resilience and social support of young adults living with mental illness in the city of Tshwane, Gauteng province, South Africa’, *Curationis* 43(1), a2084. 10.4102/curationis.v43i1.208 PMC7756966 33354974

[CIT0013] Hill, P.L., Edmonds, G.W. & Hampson, S.E., 2019, ‘A purposeful lifestyle is a healthful lifestyle: Linking sense of purpose to self-rated health through multiple health behaviours’, *Journal of Health Psychology* 24(10), 1392–1400. 10.1177/1359105317708251 28810459 PMC5665713

[CIT0014] Jekauc, D., Fiedler, J., Wunsch, K., Mülberger, L., Burkart, D., Kilgus, A. et al., 2023, ‘The effect of self-confidence on performance in sports: A meta-analysis and narrative review’, *International Review of Sport and Exercise Psychology* 1–27. 10.1080/1750984X.2023.2222376

[CIT0015] Khan, K.S., Mamun, M.A., Griffiths, M.D. & Ullah, I., 2022, ‘The mental health impact of the Covid-19 pandemic across different cohorts’, *International Journal of Mental Health and Addiction* 20(1), 380–386. 10.1007/s11469-020-00367-0 32837440 PMC7347045

[CIT0016] Kirkbride, J.B., Anglin, D.M., Colman, I., Dykxhoorn, J., Jones, P.B., Patalay, P. et al., 2024, ‘The social determinants of mental health and disorder: Evidence, prevention and recommendations’, *World Psychiatry* 23(1), 58–90. 10.1002/wps.21160 38214615 PMC10786006

[CIT0017] Konaszewski, K., Niesiobędzka, M. & Surzykiewicz, J., 2021, ‘Resilience and mental health among juveniles: Role of strategies for coping with stress’, *Health and Quality of Life Outcomes* 19(58), 1–12. 10.1186/s12955-021-01701-3 33602278 PMC7891003

[CIT0018] Kroukamp, L., 2018, ‘If you want to get rid of drugs in Tshwane stop trying to get rid of drugs’, Other thesis, Department of Family Medicine, University of Pretoria.

[CIT0019] Larson, D.B., Sherrill, K.A., Lyons, J.S., Craige, F.C., Thielman, S.B., Greenwold, M.A. et al., 1992, ‘Dimensions and valences of measures of religious commitment found in the American Journal of Psychiatry and the Archives of General Psychiatry, 1978–1989’, *American Journal of Psychiatry* 149(4), 557–559. 10.1176/ajp.149.4.557 1532477

[CIT0020] Lee, L.O., Grodstein, F., Trudel-Fitzgerald, C., James, P., Okuzono, S.S., Koga, H.K. et al., 2022, ‘Optimism, daily stressors, and emotional well-being over two decades in a cohort of aging men’, *The Journal of Gerontology Series B Psychological Sciences and Social Sciences* 77(8), 1373–1383. 10.1093/geronb/gbac025 PMC9371455 35255123

[CIT0021] Li, X., Zhao, X., Lee, H.L. & Voss, C., 2023, ‘Building responsive and resilient supply chains: Lessons from the COVID-19 disruption’, *Journal of Operations Management* 69(3), 352–358. 10.1002/joom.1250

[CIT0022] Lincoln, Y.S. & Guba, E.G., 1985, *Naturalistic inquiry*, Sage Publications, Newbury Park, CA.

[CIT0023] Liu, Q., Jiang, M., Li, S. & Yang, Y., 2021, ‘Social support, resilience, and self-esteem protect against common mental health problems in early adolescence: A nonrecursive analysis from a two-year longitudinal study’, *Medicine (Baltimore)* 100(4), e24334. 10.1097/MD.0000000000024334 33530225 PMC7850671

[CIT0024] Look, B.C. & Belaval, Y., 2024, ‘Gottfried Wilhelm Leibniz’, *Encyclopedia Britannica*, viewed 06 August 2024, from https://www.britannica.com/biography/Gottfried-Wilhelm-Leibniz.

[CIT0025] McGrath, J.J., Al-Hamzawi, A., Alonso, J., Altwaijri, Y., Andrade, L.H., Bromet, E.J. et al., 2023, ‘Age of onset and cumulative risk of mental disorders: A cross-national analysis of population surveys from 29 countries’, *The Lancet Psychiatry* 10(9), 668–681. 10.1016/S2215-0366(23)00193-1 37531964 PMC10529120

[CIT0026] Öcal, E.E., Demirtaş, Z., Atalay, B.I., Önsüz, M.F., Işikli, B., Metintaş, S. et al., 2022, ‘Relationship between mental disorders and optimism in a community-based sample of adults’, *Behavioral Sciences* 12(52), 1–10. 10.3390/bs12020052 PMC8869756 35200303

[CIT0027] Schug, C., Morawa, E., Geiser, F., Hiebel, N., Beschoner, P., Jerg-Bretzke, L. et al., 2021, ‘Social support and optimism as protective factors for mental health among 7765 healthcare workers in Germany during the COVID-19 pandemic: Results of the VOICE study’, *International Journal of Environmental Research and Public Health* 18(7), 3827. 10.3390/ijerph18073827 33917493 PMC8038794

[CIT0028] Senger, A.R., 2023, ‘Hope’s relationship with resilience and mental health during the COVID-19 pandemic’, *Current Opinion in Psychology* 50, 101559. 10.1016/j.copsyc.2023.101559 36812769 PMC9886565

[CIT0029] Shrivastava, A. & Desousa, A., 2016, ‘Resilience: A psychobiological construct for psychiatric disorders’, *Indian Journal of Psychiatry* 58(1), 38–43. 10.4103/0019-5545.174365 26985103 PMC4776579

[CIT0030] Snyder, C.R., Harris, C., Anderson, J.R., Holleran, S.A., Irving, L.M., Sigmon, S.T. et al., 1991, ‘The will and the ways: Development and validation of an individual differences measure of hope’, *Journal of Personality and Social Psychology* 60(4), 570–585. 10.1037//0022-3514.60.4.570 2037968

[CIT0031] Sorsdahl, K., Petersen, I., Myers, B., Zingela, Z., Lund, C. & Van Der Westhuizen, C., 2023‚ ‘A reflection of the current status of the mental healthcare system in South Africa’, *SSM – Mental Health* 4, 100247. 10.1016/j.ssmmh.2023.100247

[CIT0032] South African College of Applied Psychology, 2019, *The shocking state of mental health in South Africa in 2019*, viewed 13 May 2024, from https://www.sacap.edu.za/blog/management-leadership/mental-health-south-africa/.

[CIT0033] Stahl, N.A. & King, J.R., 2020, ‘Expanding approaches for research: Understanding and using trustworthiness in qualitative research’, *Journal of Development Education* 44(1), 26–28.

[CIT0034] Swan, B., 2018, ‘Faith and mental health’, *The Furrow* 69(5), 277–286.

[CIT0035] Tesch, R., 1990, *Qualitative research: Analysis types and software tools*, Falmer, New York, NY.

[CIT0036] Torales, J., Barrios, I., Melgarejo, O., Diaz, N.R., O’Higgins, M., Navarro, R. et al., 2024, ‘Hope, resilience and subjective happiness among general population of Paraguay in the post COVID-19 pandemic’, *International Journal of Social Psychiatry* 70(3), 489–497. 10.1177/00207640231216342 38059364

[CIT0037] Varkey, B., 2021, ‘Principles of clinical ethics and their application to practice’, *Medical Principles and Practice* 30(1), 17–28. 10.1159/000509119 32498071 PMC7923912

[CIT0038] World Health Organization, 2022, *Mental disorders*, viewed 13 May 2024, from https://www.who.int/news-room/fact-sheets/detail/mental-disorders.

[CIT0039] World Health Organization, 2024, *Mental health of adolescents*, viewed 01 December 2024, from https://www.who.int/news-room/fact-sheets/detail/adolescent-mental-health.

[CIT0040] Yamashita, A., Yoshioka, S.I. & Yajima, Y., 2021, ‘Resilience and related factors as predictors of relapse risk in patients with substance use disorder: A cross-sectional study’, *Substance Abuse Treatment, Prevention, and Policy* 16(40), 1–9. 10.1186/s13011-021-00377-8 33947412 PMC8097930

[CIT0041] Yanal, R.J., 1987, ‘Self-esteem’, *Noûs* 21(3), 363–379. 10.2307/2215187

